# Why “decoding the elite soccer player’s psychological profile” fails to score

**DOI:** 10.1073/pnas.2502322122

**Published:** 2025-06-06

**Authors:** Lisa Musculus, Duarte Araújo, Elisa Bisagno, Henrik Gustafsson, Anton Kalén, Magnus Lindwall, Alexandra Pérez-Ferreirós, Markus Raab, A. Mark Williams, Andreas Stenling

**Affiliations:** ^a^Department of Performance Psychology, Institute of Psychology, German Sport University Cologne, Cologne 50933, Germany; ^b^Interdisciplinary Centre for the Study of Human Performance (CIPER), Faculty of Human Kinetics, University of Lisbon, Lisbon 1499-002, Portugal; ^c^Surgical, Medical and Dental Department of Morphological Sciences related to Transplant, Oncology and Regenerative Medicine, University of Modena and Reggio Emilia, Reggio Emilia 421 21, Italy; ^d^The Department of Sport and Social Sciences, Norwegian School of Sport Sciences, Oslo 0863, Norway; ^e^Department of Educational Studies, Faculty of Arts and Social Sciences, Karlstad University, Karlstad SE-651 88, Sweden; ^f^Digital Services and Systems, Department of Computer Science, Electrical and Space Engineering, Lulea University, Luleå SE-971 87, Sweden; ^g^Department of Psychology, University of Gothenburg, Gothenburg SE-405 30, Sweden; ^h^ Independent Researcher; ^i^Department of Health and Kinesiology, College of Health, The University of Utah, Salt Lake City, UT 84112; ^j^Department of Psychology, Umeå University, Umeå SE-901 87, Sweden; ^k^Department of Sport Science and Physical Education, University of Agder, Kristiansand 4630, Norway

In their recent article, Bonetti et al. (1) claim to reveal an “ideal psychological profile” for success in elite soccer, which they argue has important implications for talent identification. While these claims are intriguing both for researchers and practitioners in soccer and potentially across sports, we strongly question whether the study’s design, data, and analyses justify such conclusions. Moreover, the conclusions also contradict a substantial body of literature in sport science and sport psychology (2–7), including recent comprehensive reviews, thereby placing further scrutiny on the accuracy of the findings and conclusions.

The authors compare elite soccer players to a control group drawn from the general population, rather than a more appropriate group involving ideally subelite, but at worst low-level soccer players. The selection of a general control group makes it impossible to determine whether between-group differences arise due to soccer expertise or simply from general variations in lifestyle factors. Consequently, the findings present no evidence to suggest that any psychological or cognitive measure offers predictive utility in distinguishing soccer players across different skill levels.

The study design is cross-sectional, so it cannot address whether executive functioning (EF) or certain personality traits cause elite performance or result from intense training and maturation. The absence of longitudinal or intervention data precludes any meaningful statement about future potential or talent identification. Published reports clearly indicate that age, training volume, and domain-specific cognitive skills are stronger predictors of expert performance than general EF tasks (2, 4).

The machine-learning analyses employed raise concerns about overfitting, given the modest sample size and numerous parameters (8, 9). Such “black-box” models may produce high accuracy within the same sample yet fail to generalize to new data. Without robust out-of-sample validation, it is premature to claim near-perfect classification of elite players.

Bonetti et al. (1) frequently emphasize the importance of their findings for talent identification and player development, yet no results directly address how personality or EF evolve over time. Valid conclusions relating to talent identification require multifactorial models that integrate technical, tactical, psychosocial, and physical measures (4, 6, 7). A single snapshot of adult players does not clarify how or whether these traits predict long-term success or provide any insights into how these measures change over time.

The methodological weaknesses of the study are examples of common problems in research on talent identification across sports (10), calling into question statements such as success in soccer is “extremely improbable” without players having the “profile” suggested by the authors. Such bold statements risk misleading stakeholders by overselling findings that are not supported by the evidence presented.

Overall, Bonetti et al.’s conclusions inappropriately go well beyond what can be concluded from the study and contradict existing state-of-the-art research in sport science and sport psychology. We call for more rigorous and appropriate designs that compare soccer players of varying performance levels, embrace a multifactorial approach, use a longitudinal design, and include more appropriate controls to substantiate any claims about talent identification and the supposedly “indispensable” psychological profile for elite soccer.
